# The Pathological Society of Philadelphia

**Published:** 1858-11

**Authors:** 


THE PATHOLOGICAL SOCIETY OF PHILADELPHIA.—At a meeting held on the 13th of October, the following officers were elected for the ensuing year:—

| | |
|---|---|
| *President,* | DR. LA ROCHE. |
| *Vice-Presidents,* | DRS. STILLÉ and EDWARD HARTSHORNE. |
| *Treasurer,* | DR. ADDINELL HEWSON. |
| *Secretary,* | DR. DA COSTA. |
| *Assistant Secretary,* | DR. J. H. BRINTON. |

